# New anesthetic programs for big elderly in day surgery

**DOI:** 10.1186/1471-2318-11-S1-A38

**Published:** 2011-08-24

**Authors:** F Oliva, G Dimarzio, M De Vizia, B Lettieri

**Affiliations:** 1 Department of Anaesthesia, Surgical and Emergency Science, Second University of Naples, Italy

## Background

Today the availability of new local anesthetics and the use of analgesics, allow the modulation of analgesia, maintaining a state of consciousness.

**Figure 1:**
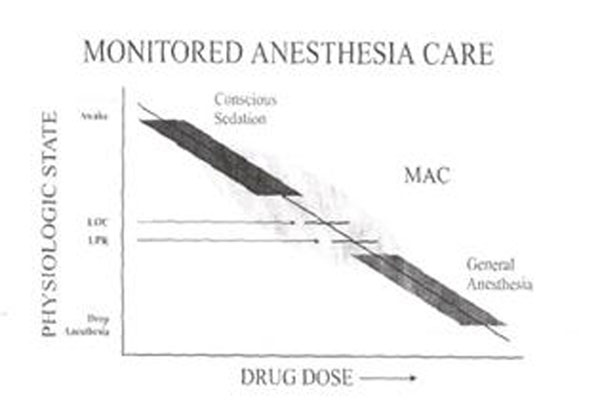
MAC is a conscious sedation, that allows analgesia and sedation**.** In fact, MAC allows the adjustment of the level of analgesia at different stages of surgery, using local anesthetics with prolonged effect and analgesics with strong action but rapid reversibility**.**

An answer to the needs of patients >75 years undergoing surgery is the technique Monitored Anesthesia Care (MAC), defined “the middle land” (*ASA 2003 – S.Francisco*) Figure 1.

## Materials and methods

A study was conducted in double-blind, random, with 86 patients subjected to two different regimens of sedation with midazolam and propofol, pain with remifentanil. Inclusion criteria: age> 70 years, body weight 69 ± 6 kg, informed consent MAC, ASA II-III stabilized cardiovascular and respiratory conditions (pO2 ≤70 and pCO2 <45 mmHg). Exclusion criteria: risk of bleeding, ASA III in acute, severe neurological disorders, severe hepatic dysfunction. During surgery, the monitoring of the level of sedation and mental status was performed comparing three methods of assessment: the clinical evaluation of drug effects on the CNS (Observer Assessment of Alertness/Sedation scale OAA/S), MAC has a sedation level of 3-4, the assessment of sedation according to Ramsay scale, and instrumental evaluation (Bispectral Index BIS). The clinical procedure was: O2 inhalation when the area was infiltrated with local anesthetic or BNP, when patients had pain a continuous infusion of remifentanil was activated: 0.025 to 0.05 mcgr/kg/m. Patients were dichotomized randomly into two groups with different sedation: group P (45 patients), starter bolus of 0.5 mg/kg propofol and continuous infusion of propofol 1-2 mg/kg/h; group M (41 patients), starter bolus of 0.03 to 0.05 mg/kg midazolam and then infusion of 1-2 μgr/kg/h. Tables 1 and 2.

**Table 1:** 

	T 10 min	T 20 min	T 30 min	T 40 min
BIS	72 (28-95)	66 (20-98)	70 (15-98)	74 (30-98)
OAA/S	4 (1-5)	3-4 (1-5)	3-4 (1-5)	4 (1-5)
Ramsay	3-4 (1-6)	4 (1-6)	3-4 (1-6)	3-4 (1-6)

media dei valori strumentali e clinici del gruppo P

**Table 2:** 

	T 10 min	T 20 min	T 30 min	T 40 min
BIS	64 (18-96)	58 (15-93)	62 (16-94)	66 (20-98)
OAA/S	4 (1-5)	3-4 (1-5)	3-4 (1-5)	3-4 (1-5)
Ramsay	3-4 (1-6)	4 (1-6)	3-4 (1-6)	3-4 (1-6)

media dei valori strumentali e clinici del gruppo M

## Results

The scale of Ramsay sedation and OAA/S showed similar results, BIS values> 70 represented a significant predictor in the study of a more rapid recovery of state of consciousness, which has favored fast tracking. One fact that emerged from the study: the 3 scores of sedation are significantly correlated (P <0.001), but this correlation is lost in the M group when the values of BIS ≤ 70 in respiratory parameters is compromised greater than in group P (P = 0.001) Table 3.

**Table 3:** 

	Midadolam	Propofol	Remifentanil
onset of sedation	fast	moderate	fast
drug effects recovery	fast	slow	fast
pain at injection	yes	no	no
intra-postoperative pain	moderate	moderate	minimal
hemodynamic depression	moderate	minimal	minimal
respiratory variations	slight desaturation (< 30 %)	minimal	minimal
ponv	minimal	minimal	minimal

## Conclusions

The observation is consistent with results of White Anesth.Analgesia '99, regarding the dose-effect curve of midazolam on the spectrum of activity of the CNS, three times higher than propofol. This leads to being more cautious in the use of midazolam in the MAC procedures.
